# In Vitro and In Vivo Antioxidative Activity against Radiation-Induced Damage and the Systematic Chemical Components of Different Extracts of *Lagotis brevituba* Maxim

**DOI:** 10.1155/2020/9726431

**Published:** 2020-12-16

**Authors:** Dan Zhang, Lihong Tan, Ling Yao, Wei Tao, Ruixue Gong, Quji LuoRong, Weiguo Cao

**Affiliations:** ^1^College of Traditional Chinese Medicine, Chongqing Medical University, Chongqing 400016, China; ^2^College of Pharmacy, Chongqing Medical and Pharmaceutical College, Chongqing 401331, China

## Abstract

*Lagotis brevituba* Maxim is a perennial species distributed in the highlands of China, which has been used for more than 2000 years as a traditional Tibetan medicinal plant. However, no attention has been paid to the antioxidant activities of *Lagotis brevituba* Maxim in vitro or in vivo. Thus, this study aimed to evaluate the in vitro and in vivo antioxidant activity of *Lagotis brevituba* Maxim against radiation-induced damage as well as the systematic chemical components. To explore the relationship between the antioxidant activity and extraction solvent, *Lagotis brevituba* Maxim was extracted with three different solvents: methanol, water, and acetone. In antioxidant assays in vitro, the water extract had the strongest reducing power, 2,2-azino-bis(3-ethylbenzothiazoline-6-sulfonic acid) diammonium salt (ABTS) radical, and 1,1-diphenyl-2-picrylhydrazyl (DPPH) radical scavenging activity compared with the methanol and acetone extracts. However, the methanol extract was more potent in the *β*-carotene/linoleic acid cooxidation assay. In antioxidant assays in vivo, mice that were exposed to 6.0 Gy^60^Co *γ*-ray whole-body radiation on day 15 after administration of *Lagotis brevituba* Maxim decreased their level of malondialdehyde (MDA) in a dose-dependent manner compared with the control group, indicating that *Lagotis brevituba* Maxim had favorable antioxidant activities in vivo. In addition, a total of 44 compounds were tentatively identified by liquid chromatography electrospray ionization quadrupole time-of-flight mass spectrometry (LC-ESI-QTOF-MS/MS), including 19 flavonoids, 14 phenols, 8 phenylethanoid glycosides, 2 iridoid glycosides, and 1 carbohydrate. We obtained 25 compounds from plants in the genus *Lagotis* for the first time. These results suggested that *Lagotis brevituba* Maxim had potent antioxidant activity and could be explored as a novel natural antioxidant.

## 1. Introduction

Free radicals are metabolic products of the human body, which exist in the human body primarily in the form of superoxide anions, hydroxyl radicals, and hydrogen peroxide radicals. Recently, increasing evidence highlighted that the overproduction of reactive oxygen species (ROS) may contribute to various diseases in the body, such as aging, diabetes, arthritis, atherosclerosis, immune disorders, cancer, inflammation, and heart disease [1–3]. To protect the human body from free radicals, many chemicals are used, such as antioxidants, including butylated hydroxyanisole (BHA), butylated hydroxytoluene (BHT), octylgallate (OG), and propyl gallate (PG) [4]. However, in recent years, many studies have shown that the excessive intake of synthetic antioxidants may be harmful to the health. For example, BHT is considered to be responsible for bladder and thyroid cancer [5]. Therefore, it is essential to develop natural nontoxic antioxidants as alternatives to synthetic ones in the food and pharmaceutical industries.

*Lagotis brevituba* Maxim (*L. brevituba* Maxim) is a perennial species distributed in the highlands of China, including Qinghai, Gansu, and Tibet. It has been used for more than 2000 years as a traditional Tibetan medicinal plant. The dried whole plant has been commonly used to treat fever, nephritis, lung disease, and hypertension as well as to relieve pain as an ancient Chinese crude drug [6]. Previous studies have shown that *L. brevituba* Maxim contains phenylethanoid glycosides, phenols, and flavonoids, which are closely related to antioxidant activity [7, 8]. However, to the best of our knowledge, no attention has been paid to the antioxidant activities of *L. brevituba* Maxim in vitro or in vivo. In order to utilize this valuable bioresource better, we desired to evaluate the antioxidant activity of *L. brevituba* Maxim systematically.

In the present study, we extracted *L. brevituba* Maxim using three different solvents, including methanol, water, and acetone. The in vitro antioxidant activities of the three different extracts were investigated using 2,2-azino-bis(3-ethylbenzothiazoline-6-sulfonic acid) diammonium salt (ABTS) radical, 1,1-diphenyl-2-picrylhydrazyl (DPPH), reducing power, and the *β-*carotene bleaching test. The potential antioxidant activity in vivo for the water extract was evaluated by measuring the changes in the activities of antioxidant enzymes in mice induced by *γ*-ray. The total phenol, flavonoid, and saponin contents of three extracts were also determined. Finally, the chemical compounds in the water extract were analyzed using liquid chromatography electrospray ionization quadrupole time-of-flight mass spectrometry (LC-ESI-QTOF-MS/MS).

## 2. Materials and Methods

### 2.1. Chemical and Plant Materials

The standards of gallic acid, rutin, and oleanolic acid were purchased from the National Institutes for Food and Drug Control (Beijing, China). The superoxide dismutase (SOD) kit, malondialdehyde (MDA) kit, and glutathione peroxidase (GSH-Px) kit were obtained from Nanjing Jiancheng Bioengineering Institute. All other chemicals and solvents were of analytical grade. The aerial parts of *L. brevituba* Maxim were collected from Aba County (Sichuan, China) in August 2017, and the samples were identified by Professor Lijuan Mei (Northwest Institute of Plateau Biology (NWIPB), Chinese Academy of Sciences, Xining, China). A voucher specimen (2014090101) was deposited in the herbarium of NWIPB, Xining. The plant materials were shade dried and ground into powder, which was sieved through a 0.8 mm metal sieve to obtain uniformly sized particles, and the material was stored at 4°C until use.

### 2.2. Extraction Procedure

We extracted 5 g of powder with methanol, water, and acetone, using an ultrasonic bath for 30 min. Then, the solvents were removed by a rotary evaporator under reduced pressure. Afterward, the obtained residues were dissolved and diluted to 25 mL with the corresponding solvent. Finally, the extracts were stored at −20°C until analysis.

### 2.3. Content Determination

#### 2.3.1. Determination of the Total Phenol Content

The Folin–Ciocalteu method was used to determine the total phenol contents of the sample extracts [9]. The absorbance was measured at 760 nm against a blank and using gallic acid as the standard. The total phenol content is expressed in mg equivalents of gallic acid/g dry weight (mg GAE/g dw.). Measurements were made in triplicate.

#### 2.3.2. Determination of the Total Flavonoid Content

The determination of the total flavonoid contents of *L. brevituba* Maxim was performed using the spectrophotometric method involving AlCl_3_ with modifications [10]. The absorbance at 510 nm was detected using a spectrophotometer, and the total flavonoid content is expressed as mg rutin equivalent (RE) per 1 g dry weight. All samples were analyzed in triplicate.

#### 2.3.3. Determination of the Total Saponin Content

The total saponin content of the extracts was determined using the vanillin-sulfuric acid method. The extracts were each mixed with 8% vanillin and 72% sulfuric acid solution and incubated at 60°C for 10 min. Then, the mixtures were cooled in an ice water bath (15 min), and the absorbances were measured at 538 nm. Oleanolic acid was used as the reference standard, and the content of total saponins is expressed as oleanolic acid equivalents (OAE g/mg dw.).

#### 2.3.4. HPLC Analysis

Extracts of L. brevituba Maxim were analyzed for the quantitative determination of echinacea glycosides and mullein glycoside by using a LC-20AD HPLC instrument (including a binary pump, a degasser, a PDA photodiode array detector and an autosampler) (Shimadzu, Kyoto, Japan). A C18 reversed-phase column (250 × 4.6 mm, 5 *μ*m i.d., SinoChrom, Dalian, China) was used at a flow rate of 1 mL/min. The injection volume was 10 *μ*L, and the column oven temperature was set to 35°C. The mobile phase consisted of (A) 0.2% aqueous formic acid and (B) acetonitrile solution, and the gradient programme was the following: 0–40 min, 13%–14% B; 40–41 min, 14%–13% B; 41–50 min, 13% B.

### 2.4. Bioactivity Studies

#### 2.4.1. Antioxidant Capacity Measurements

The analysis was performed using a Shimadzu UV-1750 spectrophotometer at the corresponding wavelength. The measurement was carried out in three stages.

*(1) 1,1-Diphenyl-2-picrylhydrazyl (DPPH) Assay*. The scavenging ability of the DPPH free radicals of the three extracts was determined using the reported methods [11,12]. DPPH (0.09 mM of methanol) (0.8 mL) was added to the extraction solution of different concentrations (0.2 mL). Afterward, the mixture was stirred and incubated for 60 min in the dark, and then the absorbance was determined at 517 nm. In the control group, ultra-pure water was used instead of the extract. Vitamin C (VC) and butylated hydroxy toluene (BHT) were used as positive controls. The half maximal inhibitory concentration (IC_50_) was calculated using the linear relationship between the compound concentration and the scavenging capacity for DPPH radicals. The percentage DPPH radical scavenging activity was calculated as follows:

(1) $$\begin{array}{c} \text{percentage of DPPH radical scavenging effect}=\left(\frac{1-{\text{OD}}_{\text{sample}}}{{\text{OD}}_{\text{control}}}\right)\times 100\%, \end{array}$$

where OD_sample_ is the absorbance of the test sample/reference compound and OD_control_ is the absorbance of the control.

*(2) 2,2-Azino-bis(3-ethylbenzothiazoline-6-sulfonic Acid) Diammonium Salt (ABTS) Assay*. Based on the method of Subhasree et al. [13], the free radical scavenging ability of plant extracts with different concentrations was determined. VC and BHT were used as positive controls and the extract without ABTS was used as a blank. ABTS^+^ were generated by the reaction of ABTS stock solution with MnO_2_. Before the assay, the freshly prepared ABTS^+^ solution was diluted with 0.01 M phosphate buffed saline (PBS, pH 7.4), and the absorbance was adjusted to 0.70 ± 0.02 at 734 nm. We added 50 microliters of each extract at different concentrations to 3 microliters of ABTS^+^ solution, incubated for 6 min at room temperature under dark conditions, and then the absorbance was measured at 734 nm. The IC_50_ values of the extracts were also calculated. The calculation results are shown as follows:

(2) $$\begin{array}{c} \text{ABTS free radical scavenging capacity }\%=\left(1-\left(\frac{{\text{OD}}_{\text{sample}}*{\text{OD}}_{\text{sample blank}}}{{\text{OD}}_{\text{control}}*{\text{OD}}_{\text{control blank}}}\right)\right)\times 100\%, \end{array}$$

where OD is the absorbance.

*(3) Reducing Power Assay*. The reduction power was determined as described by Wang et al. [14], with some modifications. We mixed 1 mL samples of different concentrations with 2 mL phosphate buffer (pH 6.8) and 2 mL potassium ferrate (1%), incubated at 50°C for 20 minutes, and then cooled immediately. Then, 2 mL trichloroacetic acid was added, shaken vigorously, centrifuged at 3000 rpm for 10 min, and finally 2 mL upper layer solution was taken. We added 2 mL ultra-pure water and 1 mL ferric chloride (0.1%), mixed well, and the absorbance at 700 nm was determined after 10 min. A higher absorbance indicates a stronger potential antioxidant capacity. VC and BHT were used as positive controls.

*(4) β-Carotene/Linoleic Acid Cooxidation Assay*. This assay is mainly suitable for the determination of the antioxidant capacity of antioxidants in an emulsified lipid system. The antioxidant activity was determined by the method of Miller et al. [15]. We placed 2 mL *β*-carotene (0.2 mg/mL) chloroform solution in a pear-shaped bottle, and 20 *μ*L linoleic acid was added and mixed with 100 mL Tavin. Then, the chloroform was removed by rotary evaporation at 40°C, and 100 mL distilled water was added to form a uniform emulsion in the blender. A portion of this reaction mixture (5 mL) was transferred into test tubes, various concentrations (0.1 mL) of the extracts were added, and the reaction mixtures were incubated for up to 2 h in a water bath at 50°C in the dark. The same procedure was repeated with BHT (the positive control) and the control in which distilled water was used instead of the extracts. The absorbance values were measured on a spectrophotometer at 470 nm. The rate of the bleaching of the *β*-carotene was monitored by measuring the absorbance at 25 min intervals. The antioxidant activity was calculated according to the following equation:

(3) $$\begin{array}{c} \text{antioxidant activity \%}=\left(1-\left(\frac{{\text{OD}}_{\text{so}}-{\text{OD}}_{\text{st}}}{{\text{OD}}_{\text{co}}-{\text{OD}}_{\text{ct}}}\right)\right)\times 100\%, \end{array}$$

where OD_st_ and OD_ct_ are the absorbances of the extract and control, respectively, at 120 min, and OD_so_ and OD_co_ are the absorbances of the extract and control, respectively, at 0 min.

#### 2.4.2. Antioxidant Determination In Vivo

*(1) Establishment of a Radiation Injury Model*. Male and female Kunming mice (14–24 g) of clean grade were provided by the Chongqing Medical University Animal Center. A total of 50 mice were used for these experiments, with 10 mice per group. Mice were randomly assigned to the following treatment groups: normal, control, model, and *L. brevituba* Maxim: H (high), M (medium), and L (low) dose (10.0, 5.0, and 2.5 g body weight/mL/day). Distilled water was orally administered to the model and normal groups. All mice except the normal control group were exposed to 6.0 Gy^60^Co *γ*-ray whole-body radiation at a dose rate of 1.01 Gy/min at a source-to-animal distance (midpoint) of 160 cm on day 15 after the administration of *L. brevituba* Maxim water extract. The animals were monitored daily for the development of symptoms of radiation sickness and mortality. All animal experiments proceeded in accordance with the international and national rules regarding animal experimentation and were ratified by the Animal Ethics Committee, Chongqing Medical University, China (no. 2017018).

*(2) Determination of SOD, MDA, and GSH-Px*. Blood samples were taken from the eyeballs of animals on the seventh day after irradiation. The mice were sacrificed to obtain the brains and livers. Organic tissues were separated and washed with saline. Accurately weighed tissue samples (1 g) and 9 mL of precooled saline were mixed with a tissue homogenizer at 10,000 r/min to prepare a 10% homogenate. The samples were then centrifuged for 10 min, and the supernatant was removed and stored at −80°C. Then, the SOD, MDA, and GSH-Px values from the blood, brains, and livers were measured according to the kit instructions.

### 2.5. LC-ESI-QTOF-MS/MS Analysis

LC-ESI-QTOF-MS/MS (AB Sciex, Framingham, MA, USA) was used in both the positive and negative ion modes. The optimum ESI operational conditions were as follows: capillary voltage, 5.5 kV (ESI+) or −5.5 kV (ESI−); source temperature, 600°C; nebulizer gas, N_2_, 55 psi; scan range, 50–1000 m/z. The injection volume was 3 *μ*L, and the total flow rate was 0.2 mL/min. We used 1% formic acid in water (A) and methanol (B) as the gradient mobile phase. The procedure was as follows: 15%–20% B; 20–45 min, 20%–40% B; 45–65 min, 40%–60% B; 65–75 min, 60%–80% B; and 75–80 min, 80%–15% B.

### 2.6. Statistical Analysis

All experiments were performed in triplicate, and all data are expressed as the mean ± standard deviation (SD). One-way analysis of variance (ANOVA) was used for the statistical analysis (SPSS 17.0, SPSS Inc., Chicago, IL, USA). Values of *P* < 0.05 were considered statistically significant.

## 3. Results and Discussion

### 3.1. Content Determination

The solubility of antioxidants in different solvents is different; therefore, choosing the appropriate solvent as the extraction medium is crucial. The contents of total phenols, total flavonoids, and saponins of the *L. brevituba* Maxim extracts in different solvents are presented in Table 1. The highest contents of phenols (38.59 mg GAE/g), flavonoids (29.29 mg RE/g), and saponins (13.63 mg OAE/g) were found in the water extracts, which indicates that there were many polar components in this plant. The solubility of the components varied with their chemical structure and solvent structure. Flavonoids and phenolic components generally have phenolic hydroxyl groups, and so they seldom exist in acetone with a lower polarity [6]. The results showed that water was the most effective solvent for extracting polyphenols from *L. brevituba* Maxim.

**Figure 1:**
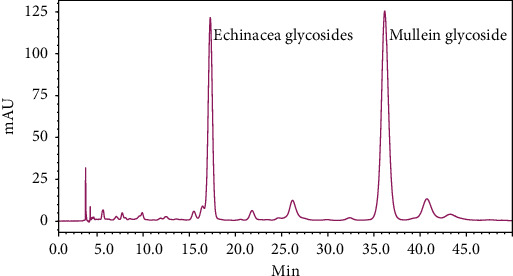
Chromatograms of echinacea glycosides and mullein glycoside in the water extracts.

Echinacea glycosides and mullein glycoside are the index components of *L. brevituba* Maxim. In order to control the quality of *L. brevituba* Maxim, the contents were determined. Based on the chromatograms, echinacea glycosides and mullein glycoside in the aqueous extracts were good separated at a retention time of 17.2 and 36.3 min, respectively (Figure 1). According to the HPLC results, the contents of echinacea glycosides and mullein glycoside were 7.34 *μ*g/mg and 9.69 *μ*g/mg, respectively.

### 3.2. Antioxidant Activity

#### 3.2.1. DPPH Radical Scavenging Activity

**Figure 2:**
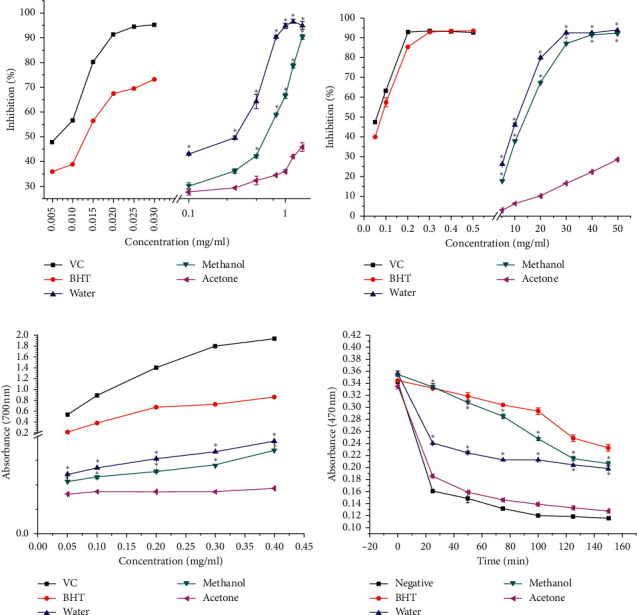
Antioxidant capacity of different solvent extracts from *L. brevituba* Maxim. (a) DPPH radical scavenging rate. (b) ABTS radical scavenging rate. (c) Ferric reducing antioxidant power. (d) *β*-Carotene/linoleic acid cooxidation activity. Values are means ± S.D. Compared with acetone, ^*∗*^*P* < 0.05.

The scavenging DPPH activities of *L. brevituba* Maxim extracts are presented in Figure 2(a). The DPPH inhibition rates increased with the concentration. The antioxidant capacities of the positive controls (BHT and VC) were higher, with IC_50_ values of 0.013 mg/mL for VC and 0.014 mg/mL for BHT, followed by water extract (0.547 mg/mL), methanol extract (0.855 mg/mL), and the acetone extract, which showed a substantially weaker activity than the other two extracts. This phenomenon maybe related to the content of phenolic compounds in the extracts. Water extracts have stronger free radical scavenging capacity as they contain more phenolic compounds. This is consistent with the previous literature: there is a highly positive correlation between total phenols and antioxidant activity [16, 17].

#### 3.2.2. ABTS Radical Scavenging Activity

The ABTS^+^ assay is an excellent tool for determining the antioxidant activity of hydrogen-donating antioxidants and chain-breaking antioxidants. As shown in Figure 1(b), the positive controls BHT and VC had the highest scavenging rates of ABTS free radical, which were close to 95% when the concentration reached 0.3 mg/mL. Similar to the DPPH scavenging activities, the *L. brevituba* Maxim extracts were not as potent as the controls, and the water and methanol extracts were noticeably more active than the acetone extract (the IC_50_ values were 12.754 mg/mL and 14.792 mg/mL, respectively). Generally, the scavenging activity of ABTS was consistent with the results of the DPPH assay, indicating that the water extract was rich in antioxidant constituents. Comparing the results of DPPH and ABTS, the IC_50_ value of the former was higher than that of the latter, which indicated that the scavenging capacity of the extract for DPPH radicals was stronger than that of the ABTS radicals.

#### 3.2.3. Ferric Reducing Antioxidant Power

A reducing power assay is an important parameter for compounds to become effective antioxidants and uses a different mechanism to explain antioxidant activity [18]. This method is based on the capacity of antioxidants to reduce Fe^3+^/ferricyanide complex to the Fe^2+^ form [19]. A higher absorbance of the reaction solution indicates a higher Fe^2+^ content and stronger reduction ability. From the results, it appears that the reduction ability increased with the increase of the concentration, and VC had the strongest reduction ability, followed by the water, methanol, and acetone extracts (Figure 1(c)). The water extract showed the best reducing power compared with the methanol and acetone extracts. We attribute this result to the fact that the water extracts contained more phenolics and flavonoids. In addition, the absorbance of VC at the lowest concentration was approximately 3.5 times that of water extract at the highest concentration. Thus, we propose that the reduction ability of the extract was lower than that of the positive control, but that the extract still provides a moderate reduction ability.

#### 3.2.4. *β*-Carotene/Linoleic Acid Cooxidation Activity

The bleaching method of *β*-carotene is based on the fact that linoleic acid in emulsion can be oxidized automatically, and the generated free radicals react with *β*-carotene to cause the yellow attenuation of *β*-carotene. However, in the presence of antioxidants, the bleaching rate of *β*-carotene is slowed, and the degree of inhibition or slowing down is related to the antioxidant activity of the substances in the system. The inhibition activities of the samples from the *β*-carotene/linoleic acid cooxidation assay are shown in Figure 1(d). In the control tube without antioxidants, the bleaching rate of *β*-carotene was very fast, and the color almost faded within 60 min. However, the absorbance value of the solution increased with *L. brevituba* Maxim extracts and BHT, but decreased with the treatment time at 470 nm, and the fading rate of *β*-carotene slowed down, which indicated that the extracts and BHT had inhibitory effects on *β*-carotene bleaching.

Similar to the results of other antioxidant assays, the acetone extract showed the lowest power; however, there was a difference between the efficacies of the water and methanol extracts, and in this case, the methanol extract was more potent than the water extract. As the reaction curve of the methanol extract was higher than that of the water extract as a whole, the methanol extract always demonstrated a stronger ability to inhibit oxidation than the water extract at each point in time. According to the previous literature, we speculate that this may be due to the fact that many hydrophilic antioxidants cannot be expressed in a lipid emulsion system [20].

#### 3.2.5. Antioxidant Assay In Vivo

MDA is the product of lipid peroxidation induced by oxygen free radicals on unsaturated fatty acids. Its content can reflect the degree of lipid peroxidation in the body and indirectly reflect the degree of damage of oxygen free radicals to cells. SOD and GSH-Px are important antioxidant enzymes in vivo. Their levels indicate the ability of scavenging free radicals [21]. Therefore, to effectively reflect the biological activity of the water extract, we chose these three indicators as biomarkers. The values of SOD, MDA, and GSH-Px in the blood and organic tissue are shown in Table 2. The results indicated that in different tissues, the levels of the three variables were not the same. Except for the MDA content in the blood in high and middle dose groups, the contents of SOD, MDA, and GSH-Px in each tissue and treatment group were significantly different from those in the control group (*P* < 0.05). This may be due to different tissues having different antioxidant abilities; therefore, tissues respond differently to oxidative stress [22, 23]. With the increasing concentration of *L. brevituba* Maxim water extract, the activities of SOD and GSH-Px increased gradually and then returned to their normal levels, while the amount of MDA decreased to the level of the control group. These results suggest that *L. brevituba* Maxim can increase the activity of antioxidant enzymes, inhibit lipid peroxidation in vivo, and reduce peroxidation damage in model mice.

### 3.3. Chemical Composition of *L. brevituba* Maxim

**Figure 3:**
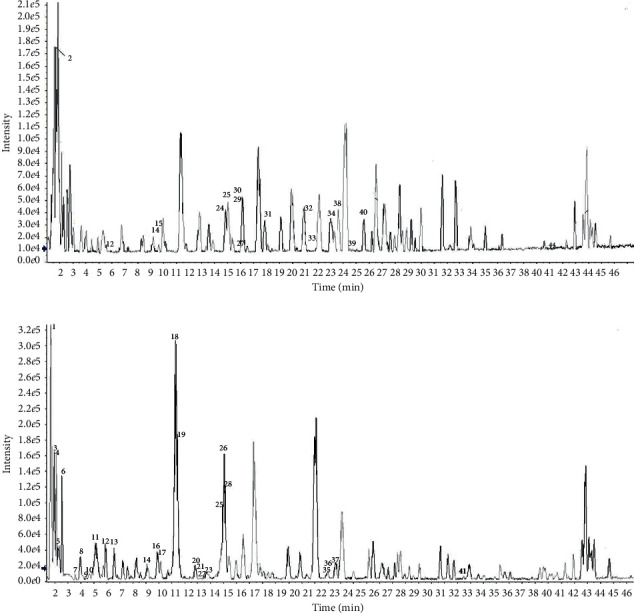
LC-ESI-QTOF-MS/MS base peak chromatograms (BPC) of *L. brevituba* Maxim extract. (a) MS in positive ion mode; (b) MS in negative ion mode.

Putative identification was performed using a detailed study of the fragmentation patterns produced by LC-ESI-QTOF-MS/MS in both the positive and negative ion modes with the water extract. Substances in the *L. brevituba* Maxim extracts were mainly present as glycosides, which were classified as phenols, flavonoids, phenylethanoids, and other glycosides. The loss of 176 daltons is indicative of glucuronide, the loss of 162 daltons is indicative of hexoses (glucose or galactose; the most common sugars found in flavonoids), the loss of 146 daltons is indicative of rhamnose, the loss of 133 daltons is indicative of pentoses (xylose or arabinose; the most common pentoses found in natural products), the loss of 308 daltons is indicative of one molecule of rhamnose plus one of hexose (rutinoside or rhamno-hexoside), and the losses of 90 and 120 daltons are indicative of C-glycoside phenolic compounds [23, 24]. The compounds in *L. brevituba* Maxim are discussed below and are summarized in Table 3 and Figure 3.

#### 3.3.1. Phenol

**Figure 4:**
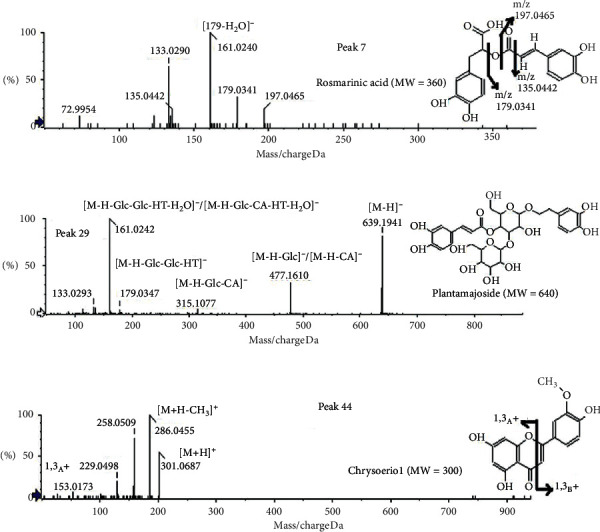
MS/MS spectra of compounds isolated from *L. brevituba* Maxim extract: (a) peak 7 and (b) peak 29 in negative mode; (c) peak 44 in positive mode.

Several phenols in the *L. brevituba* Maxim extract were tentatively identified by their fragmentation patterns and by comparing their characteristic MS fragmentation data with those reported in the literature. The identified compounds can be roughly divided into two main categories, namely, hydroxycinnamic acid derivatives and hydroxybenzoic acid derivatives. The deprotonated molecular ion [M − H]^−^ at m/z 179 at 6.57 min was indicative of caffeic acid (compound 31) [25]. The major fragment ions produced by MS/MS were at m/z 135, corresponding to the loss of CO_2_ from the carboxylic acid group with loss of H_2_O. Compound 28 and compound 27 were identified as coumaric acid ([M − H]^−^ m/z 163) and ferulic acid ([M − H]^−^ m/z 193), and they produced major fragment ions at m/z 119 and 149, respectively, for the loss of CO_2_ from their precursor ions [26].The fragmentation patterns of compounds 8 ([M − H]^−^ m/z 325) and 10 ([M − H]^−^ m/z 355) were similar to those of the aforementioned two acids. The main MS/MS ions at 163 and 193 indicated a loss of hexoside; therefore, those two compounds were identified as coumaric hexoside and ferulic acid hexoside [27]. Compound 7 was identified as rosmarinic acid [28], and the [M − H]^−^ ion at m/z 359 was indicative of the two main constituents of rosmarinic acid, namely, caffeic acid at m/z 179 and the 2-hydroxy derivative of hydrocaffeic acid at m/z 197, as illustrated in Figure 4(a).

The other ions at 161 and 135 were the same as those produced by caffeic acid. Compound 42 was identified as cinnamic acid ([M − H]^−^ m/z 147), and the ions at m/z 119 and 101 were consistent with this assignment [29]. The [M − H]^−^ ion at m/z 353 was identified as 4-caffeoylquinic acid [30], which gave MS/MS ions at m/z 191, 173, and 161. The product ion at m/z 191 was for the quinic acid part, and the ion at m/z 161 was for the caffeic acid part, which revealed that compound 9 was derived from the condensation of these two constituents. The [M − H]^−^ signals at m/z 153 and m/z 137 were identified as protocatechuic acid (compound 36) [31] and hydroxybenzoic acid (compound 12) [32], respectively, which are both hydroxybenzoic acid derivatives. Three other reference phenolic acids were also observed and identified in *L. brevituba* Maxim. The [M − H]^−^ ion at m/z 117 gave the fragment ions at m/z 99 and 73 and was identified as succinic acid (compound 39) [33]. The [M − H]^−^ ion at m/z 133 gave fragment ions at m/z 115, 71, and 59 and was identified as malic acid (compound 5) [25]. The [M − H]^−^ ion at m/z 191 gave fragment ions at m/z 111, 87, and 59 and was identified as citric acid (compound 37) [31].

#### 3.3.2. Flavonoids

Flavonoids represent another important group of metabolites that were characterized in this study [34]. The MS/MS spectra of the protonated molecules ([M + H]^+^) generated in the positive ion mode yielded the A and B series of ions characteristic of C-ring cleavage. Two kinds of flavonoids, namely, flavones and flavonols, were observed and identified in this study. For flavones, chrysoeriol, apigenin, luteolin, iridin, and their derivatives were identified in the MS/MS signals. The compound found at 40.38 min was identified as chrysoeriol (compound 44) [35], and its [M + H]^+^ ion at m/z 301 and high intensity fragment ion at m/z 286 indicated the characteristic neutral loss of CH_3_. The ion at m/z 153 is abundant in flavonoid compounds, as observed in chrysoeriol, due to the fragmentation pattern of ^1,3^A^+^ and ^1,3^B^+^, and the details are presented in Figure 4(c).

Two compounds that generated [M + H]^+^ ions at m/z 609 (compound 34) and 463 (compound 38) gave the same ion at m/z 301 and the same fragmentation pattern as was generated by chrysoeriol. Therefore, they were indicated as chrysoeriol derivatives. For compound 34, the fragment ions at m/z 463 [M + H − 146]^+^ and 301 [M + H − 146 − 162]^+^ indicated a loss of rhamnoside and a loss of hexoside, respectively; thus, this compound was identified as chrysoeriolrhamnoside hexoside, and the accurate positions of the glycosidic bonds are unknown. Similarly, compound 38 was identified as chrysoeriol hexoside. The [M − H]^−^ ion at m/z 269 gave fragment ions at m/z 117, 107, and 83 and was identified as apigenin (compound 43) [36]. The other three compounds were classified as apigenin derivatives, namely, apigenin glucuronide (compound 40) with successive fragmentation ions at m/z 447 [M + H]^+^ and 271 [M + H − glucuronide]^+^; apigenin hexoside (compound 11) with main fragmentation ions at m/z 433 [M + H]^+^ and 271 [M + H − hexoside]^−^; and apigenin rhamno-hexoside or apigenin rutinoside (compound 32) with fragment ions at m/z 579 [M + H]^+^ and 271 [M + H − [rhamno-hexoside]/rutinoside]^+^. The compound found in the negative mode at Rt 33.59 min was identified as luteolin (compound 41) based on its [M − H]^−^ ion at m/z 285 and MS/MS ions at 151 and 133 [37]. The [M + H]^+^ ion at m/z 523 was identified as iridin (compound 4), and the fragment ions at m/z 361, 346, and 272 were consistent with this assignment [38]. Kaempferol, isorhamnetin, quercetin, and their derivatives were identified from the MS/MS data. The [M + H]^+^ ion at m/z 317 gave fragment ions at m/z 302 and 274 and was identified as isorhamnetin (compound 15) [39]. The other two compounds were classified as isorhamnetin derivatives, namely, isorhamnetin hexoside (compound 18) with major fragmentation ions at m/z 479 [M + H]^+^ and 317 [M + H − hexoside]^+^ and isorhamnetin rhamno-hexoside or isorhamnetin rutinoside (compound 23) with fragment ions at m/z 625 [M + H]^+^ and 317 [M + H − [rhamno-hexoside]/rutinoside]^+^.

Compound 30 was identified as quercetin because the fragmentation pattern was characteristic of quercetin and was in accordance with previous reports [37]. Compound 21 was identified as quercetin glucuronide because the loss of glucuronide from the [M − H]^−^ ion at m/z 477 produced the fragment ion at m/z 301 [M − H − 176]^−^. Compound 14 was identified as quercetin hexoside because the loss of hexoside from the [M + H]^+^ ion at m/z 465 produced the fragment ion at m/z 303 [M − H − 162]^−^. Compounds 17, 24, 25, and 13 were identified as kaempferol glycosides [40] due to their characteristic ions at m/z 287 in positive mode or m/z 285 in negative mode and other fragment ions that were consistent with kaempferol. Next, compound 17 was identified as kaempferol hexoside because the [M − H]^−^ ion at m/z 609 produced a fragment ion at m/z 447 [M − H − 162]^−^, indicating a loss of hexoside, and the subsequent fragment ion at m/z 285 indicated another loss of hexoside. Compound 24 was identified as kaempferol hexoside because its [M+H]^+^ ion at m/z 449 produced a fragment ion at m/z 287 [M + H − 162]^+^, indicating a loss of hexoside. Compound 25 was identified as kaempferol rhamno-hexoside or kaempferol rutinoside because its [M + H]^+^ ion at m/z 595 produced a fragment ion at m/z 287 [M + H − 308]^+^, indicating a loss of rhamno-hexoside or rutinoside. Compound 13 was identified as kaempferol glucuronide because its [M + H]^+^ ion at m/z 463 produced a fragment ion at m/z 287 [M + H − 176]^+^, indicating a loss of glucuronide.

#### 3.3.3. Phenylethanoid Glycoside

In the present work, eight phenylethanoid glycosides were detected in the *L. brevituba* Maxim extracts. Based on the MS/MS data and literature reports, compound 29 was tentatively identified as plantamajoside [41], and its fragmentation pattern is shown in detail in Figure 4(b). Roughly speaking, this compound could be divided into three fragments, namely, glucose (Glc), caffeic acid (CA), and hydroxytyrosol (HT). The [M − H]^−^ ion at m/z 639 produced a fragment ion at m/z 477, indicating a loss of Glc or CA; the fragment ion at m/z 315 indicated the loss of both Glc and CA; the fragment ion at m/z 179 indicated the loss of two Glc units and a HT moiety; and the fragment ion at m/z 161 was formed by the loss of Glc, CA, HT, and a molecule of H_2_O or the direct loss of H_2_O from the m/z 179 ion.

This fragmentation pattern was common in phenylethanoid glycoside and ions such as m/z 315, 179, 161, and 133, and the loss of 162 is typical for this type of substance. Therefore, compound 16 was identified as plantamajoside hexoside; its [M − H]− ion at m/z 801 and the fragment ion at m/z 639 indicated a loss of hexoside. Compound 26 was identified as acteoside or isoacteoside [42], and compounds 19, 10, and 6 were identified as acteoside derivatives, namely, desrhamnosylacteoside (compound 19), methyl acteoside (compound 10), and acetyl acteoside (compound 6) [43]. In addition, compounds 33 and 22 were determined to be echinacoside [42] and lavandulifolioside [41].

#### 3.3.4. Other Compounds

In addition to the abovementioned compounds identified in this work, we characterized one carbohydrate and two iridoid glycosides. According to their fragmentation profiles, compound 1 was identified as sucrose [44], and compounds 3 and 20, which gave [M − H]^−^ ions at 373 and 509, respectively, were determined to be geniposidic acid [45] and globularimin or globularinin [32].

## 4. Conclusion

This study comprehensively estimated the antioxidative activity of different extracts and characterized the constituents of *L. brevituba* Maxim extracts. The results indicated that the water extract had the highest contents of phenols, flavonoids, and saponins and the strongest antioxidant capacity in vitro in the DPPH assay, ABTS assay, and reducing power assay. However, the *β*-carotene/linoleic acid cooxidation assay showed that the methanol extract was the most effective for the lipophilic system. The water extract of *L. brevituba* Maxim was able to restore the levels of SOD, MDA, and GSH-Px to normality and protect mice from radiation injury in vivo. In addition, a total of 44 compounds were tentatively identified by LC-ESI-QTOF-MS/MS, including 19 flavonoids, 14 phenols, 8 phenylethanoid glycosides, 2 iridoid glycosides, and 1 carbohydrate, and 25 compounds were obtained for the first time from plants in the genus *Lagotis*. Thus, we recommend further research be conducted to isolate, identify, and characterize the bioactive compounds that are responsible for the activities. The results also suggested that *L. brevituba* Maxim has considerable antioxidant activities and could be utilized as a new natural antioxidant in food and therapeutics.

## Figures and Tables

**Table 1:** Total phenol, flavonoid, and saponin contents of *L. brevituba* Maxim extracts.

Solvents	Total phenols (GAE mg/g DW)	Total flavonoids (RE mg/g DW)	Total saponins (OAE mg/g DW)
Water	38.59 ± 0.70^c^	29.29 ± 0.16^c^	13.63 ± 0.01^c^
Methanol	28.43 ± 0.05^b^	27.45 ± 0.82^b^	12.75 ± 0.06^b^
Acetone	8.18 ± 0.04^a^	6.56 ± 0.11^a^	6.96 ± 0.03^a^

Values are expressed as mean ± SEM; different superscript lowercase letters denote statistically significant difference (*P* < 0.05).

**Table 2:** Effects of *L. brevituba* Maxim extract on the level of MDA and antioxidant enzyme activities of SOD and GSH-Px^A^.

Groups	SOD			MDA			GSH-Px		
	Brain (U/mg)	Liver (U/mg)	Blood (U/ml)	Brain (nmol/mg)	Liver (nmol/mg)	Blood (nmol/mg)	Brain (U)	Liver (U)	Blood (U)
Control	167.93 ± 4.70^e^	169.00 ± 5.73^e^	127.30 ± 4.33^d^	0.49 ± 0.03^e^	0.45 ± 0.02^e^	2.15 ± 0.19^e^	56.44 ± 0.70^e^	539.44 ± 26.02^e^	338.13 ± 17.97^d^
Model	81.17 ± 2.46^a^	67.83 ± 1.87^a^	50.72 ± 1.63^a^	1.05 ± 0.06^a^	0.77 ± 0.04^a^	3.36 ± 0.42^a^	12.41 ± 0.98^a^	307.72 ± 5.99^a^	105.04 ± 2.49^a^
Low-dose	95.27 ± 3.40^b^	79.24 ± 2.43^b^	96.1 ± 3.27^b^	0.96 ± 0.02^b^	0.65 ± 0.03^b^	2.78 ± 0.07^b^	17.12 ± 1.15^b^	415.20 ± 7.65^b^	125.18 ± 4.32^a^
Medium-dose	116.53 ± 5.48^c^	102.02 ± 2.58^c^	110.28 ± 3.27^c^	0.81 ± 0.02^c^	0.57 ± 0.01^c^	2.54 ± 0.14^e^	22.88 ± 1.96^c^	450.99 ± 16.04^c^	211.51 ± 11.42^b^
High-dose	133.56 ± 3.91^d^	115.60 ± 5.32^d^	115.01 ± 1.54^c^	0.62 ± 0.03^d^	0.53 ± 0.02^d^	2.36 ± 0.06^e^	34.31 ± 1.40^d^	468.27 ± 14.57^d^	299.29 ± 23.77^c^

Values are expressed as mean ± standard deviation; different superscript lowercase letters denote statistically significant difference *P* < 0.05.

**Table 3:** Identiﬁcation of the compounds in the extract of *L. brevituba* Maxim by LC-ESI-QTOF-MS/MS.

Peak no.	Rt (min)	Molecular formula	[M + H]^+^ (m/z)	[M − H]^−^ (m/z)	Error (ppm)	MS/MS fragments	Proposed compound	Classification
1	1.69	C_12_H_22_O_11_	—	341.10894	−0.7	179.0560, 119.0353, 89.0256^a^	Sucrose	Carbohydrate
2	1.69	C_21_H_16_O_6_	365.10196	—	8.6	347.0926, 275.0721, 203.0522, 185.0411	Xanthotoxol hexoside	Phenol
3	1.99	C_16_H_22_O_10_	—	373.11402	−1.9	211.0950, 123.0450^a^	Geniposidic acid	Iridoid glycoside
4	24.59	C_24_H_26_O_13_	523.14462	—	−1.2	361.0897, 346.0666, 272.0664	Iridin	Flavonoid
5	2.34	C_4_H_6_O_5_	—	133.01425	1	115.0031, 71.0138, 59.0167^a^	Malic acid	Phenol
6	23.5	C_31_H_38_O_16_	—	665.20871	0.5	461.1660, 161.0240, 133.0290^a^	Acetyl acteoside	Phenylethanoid glycoside
7	23.31	C_18_H_16_O_8_	—	359.07724	−1.5	197.0465, 179.0341, 161.0240, 135.0442^a^	Rosmarinic acid	Phenol
8	3.93	C_15_H_18_O_8_	—	325.09289	−0.9	163.0396, 119.0503^a^	Coumaric hexoside	Phenol
9	4.42	C_16_H_18_O_9_	—	353.08781	−1.9	191.0556, 173.0459, 161.0243, 85.0292, 59.0148^a^	4-Caffeoylquinic acid	Phenol
10	23.26	C_30_H_38_O_15_	—	637.21379	1	461.1652, 443.1579, 315.1072, 193.0498, 175.0394, 153.0549^a^	Methyl acteoside	Phenylethanoid glycoside
11	21.81	C_21_H_20_O_10_	433.11292	—	−0.9	271.0593, 153.0171	Apigenin hexoside	Flavonoid
12	5.92	C_7_H_6_O_3_	139.03897	137.02442	1	93.0356, 65.0412^a^	Hydroxybenzoic acid	Phenol
13	17.94	C_21_H_18_O_12_	463.0871	461.07255	−2.4	287.0542, 241.0463, 153.0175, 285.0399^a^	Kaempferol glucuronide	Flavonoid
14	9.11	C_21_H_20_O_12_	465.10275	463.0882	0.2	303.0498, 285.0393, 301.0347, 300.0264^a^	Quercetin hexoside	Flavonoid
15	16.09	C_16_H_12_O_7_	317.06558	—	−0.7	302.0413, 274.0464	Isorhamnetin	Flavonoid
16	9.91	C_35_H_46_O_21_	—	801.24588	−0.1	639.2160, 477.1629, 315.1079, 161.0248, 133.0294^a^	Plantamajoside hexoside	Phenylethanoid glycoside
17	10	C_27_H_30_O_16_	—	609.14611	0.2	447.0840, 285.0384, 284.0320^a^	Kaempferol dihexoside	Flavonoid
18	16.09	C_22_H_22_O_12_	479.1184	—	−0.8	317.0648, 302.0413, 245.0444	Isorhamnetin hexoside	Flavonoid
19	15.96	C_23_H_26_O_11_	—	477.14024	−1	315.0501, 179.0354, 161.0246, 133.0259^a^	Desrhamnosyl acteoside	Phenylethanoid glycoside
20	12.83	C_24_H_30_O_12_	—	509.16645	−0.6	147.0443^a^	Globularimin/globularinin	Iridoid glycoside
21	12.89	C_21_H_18_O_13_	—	477.06746	−0.2	343.0440, 301.0339, 300.0275, 255.0259^a^	Quercetin glucuronide	Flavonoid
22	13.51	C_34_H_44_O_19_	—	755.2404	0.9	623.1966, 593.2107, 575.1989, 461.1668, 447.1503, 315.1076, 161.0245, 153.0550, 133.0291^a^	Lavandulifolioside	Phenylethanoid glycoside
23	15.94	C_28_H_32_O_16_	625.17631	—	−0.9	317.0642, 302.0415	Isorhamnetinrhamno-hexoside/Isorhamnetin rutinoside	Flavonoid
24	14.86	C_21_H_20_O_11_	449.10784	—	−1	287.0543, 241.0490, 153.0184	Kaempferol hexoside	Flavonoid
25	15.01	C_27_H_30_O_15_	595.16575	593.15119	−0.9	287.0536, 447.0931, 285.0389, 284.0305^a^	Kaempferol rhamno-hexoside/Kaempferol rutinoside	Flavonoid
26	15.05	C_29_H_36_O_15_	—	623.19814	0.1	461.1660, 161.0248, 135.0452^a^	Acteoside/Isoacteoside	Phenylethanoid glycoside
27	13.7	C_10_H_10_O_4_	—	193.05063	0.8	178.0264, 149.0616, 134.0371^a^	Ferulic acid	Phenol
28	11.5	C_9_H_8_O_3_	—	163.04007	0.3	119.0508, 93.0359^a^	Coumaric acid	Phenol
29	11.3	C_29_H_36_O_16_	—	639.19306	0.5	477.1610, 315.1077, 179.0347, 161.0242, 135.0442, 133.0293^a^	Plantamajoside	Phenylethanoid glycoside
30	9.12	C_15_H_10_O_7_	303.04993	301.03538	−0.4	285.0383, 229.0465255.0319^a^	Quercetin	Flavonoid
31	6.57	C_9_H_8_O_4_	—	179.03498	0.7	135.0448, 89.0388^a^	Caffeic acid	Phenol
32	21.3	C_27_H_30_O_14_	579.17083	—	−1.2	271.0593, 153.0211	Apigeninrhamno-hexoside/Apigenin rutinoside	Flavonoid
33	5.16	C_35_H_46_O_20_	—	785.25097	0.8	623.2213, 179.0357, 161.0247, 133.0292^a^	Echinacoside	Phenylethanoid glycoside
34	23.18	C_28_H_32_O_15_	609.1814	—	−1.9	463.1253, 301.0696, 286.0457, 258.0505, 229.0469, 153.0168	Chrysoeriolrhamnoside hexoside	Flavonoid
35	4.62	C_16_H_20_O_9_	—	355.10346	−2.7	193.0507, 134.0373^a^	Ferulic acid hexoside	Phenol
36	3.58	C_7_H_6_O_4_	—	153.01933	1.5	109.0297, 108.0218, 91.0189, 81.0358, 65.0032, 53.0422^a^	Protocatechuic acid	Phenol
37	2.52	C_6_H_8_O_7_	—	191.01973	−0.3	111.0085, 87.0075, 59.0149^a^	Citric acid	Phenol
38	23.64	C_22_H_22_O_11_	463.12349	—	−0.6	301.0705, 286.0465, 229.0493	Chrysoeriol hexoside	Flavonoid
39	2.22	C_4_H_6_O_4_	—	117.01933	0.5	99.9251, 73.0305^a^	Succinic acid	Phenol
40	25.51	C_21_H_18_O_11_	447.09219	—	−1.8	271.0591, 229.0486, 153.0186	Apigenin glucuronide	Flavonoid
41	33.59	C_15_H_10_O_6_	—	285.04046	−2	285.0400, 151.0034, 133.0296^a^	Luteolin	Flavonoid
42	33.89	C_9_H_8_O_2_	—	147.04515	2.4	119.0541, 103.0557, 101.0410, 77.0392, 61.9906^a^	Cinnamic acid	Phenol
43	39.27	C_15_H_10_O_5_	—	269.04555	−3.3	117.0343, 107.0136, 83.0119^a^	Apigenin	Flavonoid
44	40.38	C_16_H_12_O_6_	301.07066	299.05611	−0.2	286.0455, 258.0509, 229.0498, 153.0173, 284.0321, 256.0362, 227.00328^a^	Chrysoeriol	Flavonoid

^a^Fragmentation pattern in negative ionization mode.

## Data Availability

The data used to support the findings of this study are available from the corresponding author upon request.
